# Gene Expression and Yeast Two-Hybrid Studies of 1R-MYB Transcription Factor Mediating Drought Stress Response in Chickpea (*Cicer arietinum* L.)

**DOI:** 10.3389/fpls.2015.01117

**Published:** 2015-12-24

**Authors:** Abirami Ramalingam, Himabindu Kudapa, Lekha T. Pazhamala, Vanika Garg, Rajeev K. Varshney

**Affiliations:** ^1^International Crops Research Institute for the Semi-Arid Tropics (ICRISAT)Hyderabad, India; ^2^School of Plant Biology and Institute of Agriculture, The University of Western AustraliaCrawley, WA, Australia

**Keywords:** protein-protein interactions, chickpea, transcription factor, drought, stress tolerance, signaling pathways

## Abstract

Drought stress has been one of the serious constraints affecting chickpea productivity to a great extent. Genomics-assisted breeding has a potential to accelerate breeding precisely and efficiently. In order to do so, understanding the molecular mechanisms for drought tolerance and identification of candidate genes are crucial. Transcription factors (TFs) have important roles in the regulation of plant stress related genes. In this context, quantitative real time-PCR (qRT-PCR) was used to study the differential gene expression of selected TFs, identified from large-scale expressed sequence tags (ESTs) analysis, in contrasting drought responsive genotypes. Root tissues of ICC 4958 (tolerant), ICC 1882 (sensitive), JG 11 (elite), and JG 11+ (introgression line) were used for the study. Subsequently, a candidate single repeat MYB (*1R-MYB*) transcript that was remarkably induced in the drought tolerant genotypes under drought stress was cloned (coding sequence region for the 1R-MYB protein) and subjected to yeast two-hybrid (Y2H) analysis. The screening of a root cDNA library with Y2H using the 1R-MYB bait protein, identified three CDS encoding peptides namely, galactinol-sucrose galactosyltransferase 2, CBL (Calcineurin B-like)-interacting serine/threonine-protein kinase 25, and ABA responsive 17-like, which were confirmed by co-transformation in yeast. These findings provide preliminary insights into the ability of this 1R-MYB transcription factor to co-regulate drought tolerance mechanism in chickpea.

## Introduction

Chickpea (*Cicer arietinum* L.) is an important legume crop, which ranks third among the pulses in production globally (FAO, 2014). It is a protein rich (19–30%) crop grown widely in more than 50 countries including South East Asia, Middle East, and the Mediterranean. Chickpea has a diploid genome (2*x* = 2*n* = 16) for which the draft genome sequence (738Mb) and transcriptome assembly are available (Varshney et al., 2013a; Kudapa et al., 2014). Majority of the chickpea growing areas fall under the arid and semi-arid tropics, which are severely affected by terminal drought stress causing huge yield losses.

Drought tolerance is an intricate trait with limited successes for introgression in breeding through conventional approaches in the past. However, genomics-assisted breeding (GAB) approaches have a potential to accelerate breeding efforts in a precise and efficient manner (Varshney et al., 2005). For instance, marker-assisted backcrossing has been able to develop superior lines with enhanced root traits and higher yield under rainfed conditions (Varshney et al., 2013b, 2015). This could be possible because of the recent advances in chickpea genomics that have provided vital information for gene discovery and marker development (Garg et al., 2011; Kudapa et al., 2013; Varshney et al., 2013a). Functional genomics approaches were also used to enhance the understanding of abiotic stress responses particularly for drought and salinity in chickpea (Varshney et al., 2009; Mantri et al., 2010; Hiremath et al., 2011).

Transcription factors (TFs) are known to be the master regulators of gene expression in response to environmental stresses. TFs such as AP2/ERF, AREB/ABF, bZIP, DREB, MYC/MYB, and WRKY have been shown to play important roles in regulating abiotic stress responses (Nakashima et al., 2009; Agarwal and Jha, 2010; Hossain et al., 2010; Mizoi et al., 2012; Niu et al., 2012). Various genome-wide studies have been carried out both in model and crop plants to investigate the role of TFs in abiotic stress response mechanisms (Le et al., 2011; Katiyar et al., 2012). Strong transcriptional remodeling of chickpea roots in response to drought stress have been demonstrated in various biological processes such as signal transduction, transcription regulation, osmolyte accumulation, and reactive oxygen species (ROS) scavenging (Molina et al., 2008). Although, these studies provided some light on the role of TFs in the stress responsive signaling pathways, there is limited knowledge with respect to their function in drought tolerance in legume crops.

The above information has prompted us to study the role of TFs for drought tolerance mechanisms in chickpea. We have performed differential expression analysis of selected TFs identified through large scale expressed sequence tags (EST) analysis (Hiremath et al., 2011). The *1R-MYB* transcript which was highly up-regulated in the tolerant chickpea genotypes, was cloned (protein coding sequence) and subjected to protein-protein interaction (PPI) analysis using Y2H system to identify potential interactors/co-activators, involved in the regulation of chickpea drought tolerance mechanism. To our knowledge, this is the first report of 1R-MYB and its putative interactors for drought tolerance in chickpea.

## Materials and methods

### Plant material and stress treatment

A total of four chickpea genotypes were used for conducting expression profiling of selected TFs. Two chickpea genotypes, ICC 4958 (tolerant) and ICC 1882 (sensitive) were used as representatives of the two phenotypic categories. In addition JG 11, an elite drought tolerant cultivar and an introgression line (JG 11+) that was developed by introgressing a QTL hot-spot from ICC 4958 into JG 11 (ICC 4958 × JG 11) (Varshney et al., 2013b), were used for analysis.

Slow drought stress was imposed on the four genotypes under greenhouse conditions as described by Ray and Sinclair (1998). Root samples of the selected genotypes were collected when the transpiration ratio reached 0.1 along with their respective controls. Each treatment was maintained in three biological replications. Root tissues were collected and stored at −80°C until RNA extraction.

### RNA isolation

Total RNA was isolated from the harvested root tissues (both control and stressed samples) using TRIzol (Invitrogen, USA) reagent according to the manufacturer's protocol. Quality of all the samples was assessed on 1.2% formaldehyde agarose gel, while quantification was done by measuring A260/A280 ratio in Nanovue. First strand cDNA was synthesized from the total RNA (2.5 μg), using cDNA synthesis kit (Superscript® III, Invitrogen, USA) following manufacturer's instructions.

### Quantitative real time PCR (qRT-PCR)

cDNA samples were normalized with the house keeping gene “Glyceraldehyde 3-phosphate dehydrogenase” (*GAPDH*; Garg et al., 2010). Specific primers (Supplementary Table 1) for qRT-PCR were designed from Tentative Unique Sequences (TUSs) of selected eight TFs (from large scale EST analysis) using Primer Express software (Applied Biosystems, CA, USA). The transcript/ gene IDs of TUSs used in the study are available at Legume Information System (LIS) database (Hiremath et al., 2011; http://cicar.comparative-legumes.org/data/2011/58da8857f0f21afded122214cd604b9f/transcript_contigs.fa.gz). qRT-PCR assays were performed using Applied Biosystems 7500 Real Time PCR detection system with the SYBR green chemistry (Applied Biosystems, USA) according to the manufacturer's instructions. Three biological replications were considered for the study in order to calculate the mean relative expression levels. To determine the expression patterns, 2^−ΔΔCT^ method was employed and “*t*” test was used to calculate significances for selected genes (Livak and Schmittgen, 2001).

### Computational analysis and predictive modeling

The *MYB TF* (TUS38128) was searched against the Plant Transcription Factor Database with an *e*-value of 1E-05 and query coverage of 80% to retrieve the MYB superfamily protein sequences in chickpea. The sequences were further scanned for the conserved MYB domain using “Pfam.” Additionally, orthologs of TUS38128 were examined in some other legumes such as lotus (*Lotus japonicus*), medicago (*Medicago truncatula*), and common bean (*Phaseolus vulgaris*), using BLASTP with an e-value cut off of 1E-10 and sequence identity of ≥75%.

The protein structure of the 1R-MYB TF was predicted with iterative threading assembly refinement (I-Tasser, http://zhanglab.ccmb.med.umich.edu/I-TASSER/, last accessed 12th June 2014) (Roy et al., 2010). Using *ab initio* prediction, the models were generated based on C-score values (between −5 and 2), where a higher C-score value signifies a model with greater certainty. The three-dimensional (3D) models generated were further analyzed using Deepview/Swiss PDB Viewer v4.1.0 (http://spdbv.vital-it.ch).

### Cloning of the *1R-MYB* TF CDS into Yeast bait plasmid

First strand cDNA was synthesized using RNA extracted from the drought stressed roots of ICC 4958 (tolerant) according to the “First-Strand cDNA Synthesis” protocol (Invitrogen, USA) using 2 μg of DNAase free RNA. The coding sequence (CDS) region encoding the *1R-MYB* protein (Genbank accession: XM_004508882) with restriction sites attached, was amplified (primer pair sequences provided in Supplementary Table 1) from single stranded cDNA based on the PCR cycles and conditions described in Ziemann et al. (2008). For ligation, the pGBKT7 vector (2.5 μg) was double digested (EcoR1 and BamH1) and gel purified. The purified PCR product (150 ng) was ligated with 50 ng of pGBKT7 vector using the 5 × In-Fusion® HD Enzyme Premix, containing the “In-Fusion Enzyme.” Five microliters of the ligated product was transformed into 100 μl of Stellar™ Competent Cells (Clontech, USA) and selected on LB plates with Kanamycin (Kan; 50 μg/ml). Colonies were picked and inoculated into 5 ml LB/Kan broth and grown overnight with shaking at 37°C. Plasmids were extracted from these cultures using a purification kit (NucleoSpin® Plasmid, Macherey-Nagel, Germany) and screened for the presence of inserts with restriction digestion. To confirm the successful cloning of the *1R-MYB* CDS, the pGBKT7 vectors containing inserts were sequenced using CDS specific primers (Eurofins, India). The confirmed *1R-MYB* clone was selected and transformed into competent *S. cereviceae* Y2HGold using a high-efficiency polyethylene glycol (PEG)/LiAc-based method (Yeastmaker™ Yeast Transformation System 2 User Manual, Clontech, USA). Transformed yeast cells were selected on the minimal YSD medium deficient in TRP (SD/-W).

### Generation of drought stressed root cDNA library

The cDNA library was constructed from the roots of the drought tolerant chickpea genotype, ICC 4958 in *S. cereviceae* Y187α using Make Your Own “Mate and Plate™” Library System (Clontech, USA) following the manufacturers' instructions. Equal amounts of double stranded cDNA (3 μg) and “prey” library vector (3 μg; pGADT7-Rec) were mixed for the homologous recombination-mediated cloning using the library-scale transformation protocol (Yeast Transformation System 2 Manual, Clontech, USA). After 4 days of incubation, all the colonies were harvested in freezing medium (YPDA in 25% glycerol) and stored in aliquots at –80°C.

### Y2H assay

An aliquot (1 ml) containing >2 × 10^7^ cells of the harvested *S. cereviceae* 187α strain (harboring library constructs in pGADT7-Rec) was mated with 4–5 ml (>1 × 10^8^ cells per ml in SD/-W) of *S. cereviceae* Y2HGold (containing the *1R-MYB* constructs in pGBKT7) based on the Matchmaker™ Gold Y2H (Clontech, USA) manual. The re-suspended cells in YPDA/Kan were spread on the selective media [double dropouts (YSD-W-L)/X-α-Gal/Aureobasidin (DDO/X/A)] and incubated at 30°C for 3–5 days. Positive and negative control matings were then carried out as per the Matchmaker™ Gold Y2H manual and plated on DDO and DDO/X/A media. Single colonies were patched on QDO/X/A, followed by incubation at 30°C for 3–5 days. Yeast colony PCR using 5′ and 3′ PCR primers (Supplementary Table 1), were performed on the blue colonies identified on the QDO/X/A media to determine the presence of inserts in the prey, pGADT7-Rec clones.

Following this, plasmids were isolated from yeast colonies picked from the QDO/X/A selective media using the Easy Yeast Plasmid Isolation kit (Clontech, USA), and the “prey” vectors containing inserts of candidate interactors, were isolated by transforming into Stellar™ Competent Cells and plating on LB with ampicillin (Amp), (selective for only pGADT7-Rec clones). Colonies were picked, cultured in LB/Amp (overnight) and the plasmids were purified. The PPIs were confirmed by co-transforming *S. cerevicieae* Y2HGold with the “bait” (*1R-MYB* in pGBKT7) clone together with the interactor “prey” clone (in pGADT7-Rec) and plated on QDO/X/A. To check for any false positive interactions, the empty “bait” vector was co-transformed with the interactor prey clone and plated as above. The pGADT7-Rec clones were sequenced in the forward and reverse directions using T7 and 3′AD primers (Eurofins, India). The sequences of the identified interactors were subjected to BLASTN and BLASTX (NCBI, http://www.ncbi.nlm.nih.gov/) analyses for identification and confirming the correct orientation of the interactor sequences and to rule out any false-positive or large ORFs in the wrong reading frame.

### Determination of PPI strengths for the 1R-MYB TF

#### Biochemical analysis

The strength of interaction was determined by the growth and color of the *S. cerevisiae* Y2HGold yeast cells expressing the co-transformed bait and prey interacting proteins on QDO/X plates with increasing concentrations (0, 1, 2, 5, 10, 15, and 20 mM) of 3-AT (3-Amino-1, 2, 4 triazole). 3-AT is a competitive inhibitor of the *HIS3*-gene expression.

#### qRT-PCR analysis

*S. cerevisiae* Y2HGold cells expressing the co-transformed bait and prey interacting proteins, were grown in liquid minimal media (QDO) and harvested when the OD_600_ reached 0.5 by centrifuging at 5000 rpm. The pellets were snap-frozen in liquid nitrogen until RNA isolation. The total RNA was isolated using RNAeasy Mini Kit (Qiagen, Hilden, Germany) following the manufacturer's instructions. The DNase-treated total RNA isolated from the yeast cells, were used for synthesizing cDNA using SuperScript® III First-Strand Synthesis SuperMix (Invitrogen™, USA). The strength of the interactions, PPI-1, PPI-4, and PPI-5, were determined by qRT-PCR using two reporter genes *HIS3* and *ADE2* (Maier et al., 2012). The primer sequences used for the amplification of *HIS3* and *ADE2* along with two internal controls (TAF10 and UBC6) have been provided in Supplementary Table 1. The qRT-PCR reactions were conducted using SYBR green chemistry (Applied Biosystems, USA) in two technical replicates and two biological replicates.

## Results

### Identification of drought-responsive TF

**Figure 1:**
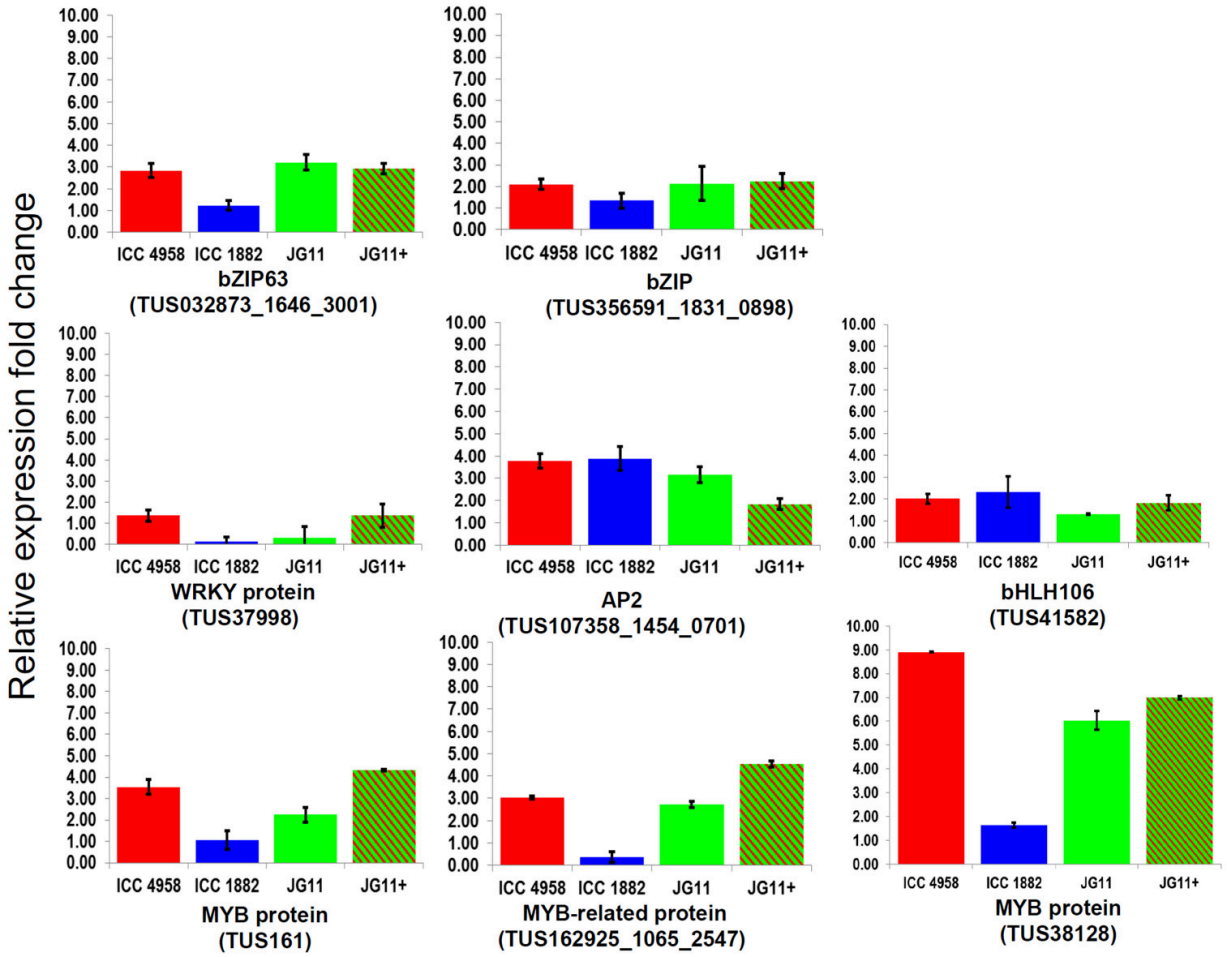
**The relative expression ratio of 8 candidate ***TF*** genes analyzed using qRT-PCR under drought stress**. The relative expression ratio of each gene was calculated relative to its expression in control sample. *GAPDH* was used as an internal control to normalize the data. The error bars representing standard deviation were calculated based on three biological and two technical replicates.

Eight TF genes, belonging to five major classes *viz., bZIP* (2), *WRKY* (1), *AP2* (1), *basic helix loop helix* (1), and *MYB* (3) were selected for quantitative expression analysis using qRT-PCR. These genes were selected from a set of 75 TUSs identified as TFs by *in silico* analysis from the 2823 differentially expressed TUSs of the chickpea transcriptome assembly generated previously (Hiremath et al., 2011). The qRT-PCR analysis revealed wide variations in the expression patterns of all the eight TFs in selected chickpea genotypes in response to drought stress (Figure 1). Among the eight TF genes, two *bZIP* genes (TUS: 032873_1646_3001 and TUS356591_1831_0898) showed ≥2 fold expression in the drought tolerant genotypes in comparison to the sensitive genotype, unlike the three TFs, *WRKY, AP2*, and *bHLH106*. These three TFs showed either ≥2 or < 2 fold expression in both the drought tolerant and the sensitive genotypes. The three *MYB* TFs (TUS:161, 162925_1065_2547, and 38128) showed ≥3 fold expression in the drought tolerant genotypes and 2 fold expression in the sensitive genotype. Noteworthy, TUS38128 was found to be highly expressed in tolerant genotypes (ICC 4958, 8.9 fold and JG 11, 6.03 fold) and in the introgression line (JG 11+, 6.99 fold) when compared to the sensitive genotype (ICC 1882, 1.6 fold). Therefore, TUS38128 was finally selected for further analyses using bioinformatics and PPI to identify putative interacting proteins using Y2H.

### Identification of MYB TFs in chickpea and related legumes

In order to understand the significance and relevance of the identified MYB-related TF under drought stress in chickpea and other related legumes, we have looked into the orthologous genes in the model legumes, lotus, and medicago, and one drought-tolerant legume, common bean. Using TUS38128 as the query sequence, 206 homologous genes belonging to MYB protein family were identified in chickpea. These genes were further classified based on the number of repeat units at the MYB-domain, into 1R-MYB/MYB-related (single repeat unit), R2R3-MYB (two repeat units), and 3R-MYB (three repeat units; Dubos et al., 2010). A total of 125 homologs including TUS38128, which contained only one repeat unit in their encoded proteins were classified as *1R-MYB*/*MYB*-related genes in chickpea. The orthologous relationship in common bean, lotus, and medicago for TUS38128 is represented as a Circos plot (Krzywinski et al., 2009; Supplementary Figure 1).

### Prediction of protein structure for the 1R-MYB TF

**Figure 2:**
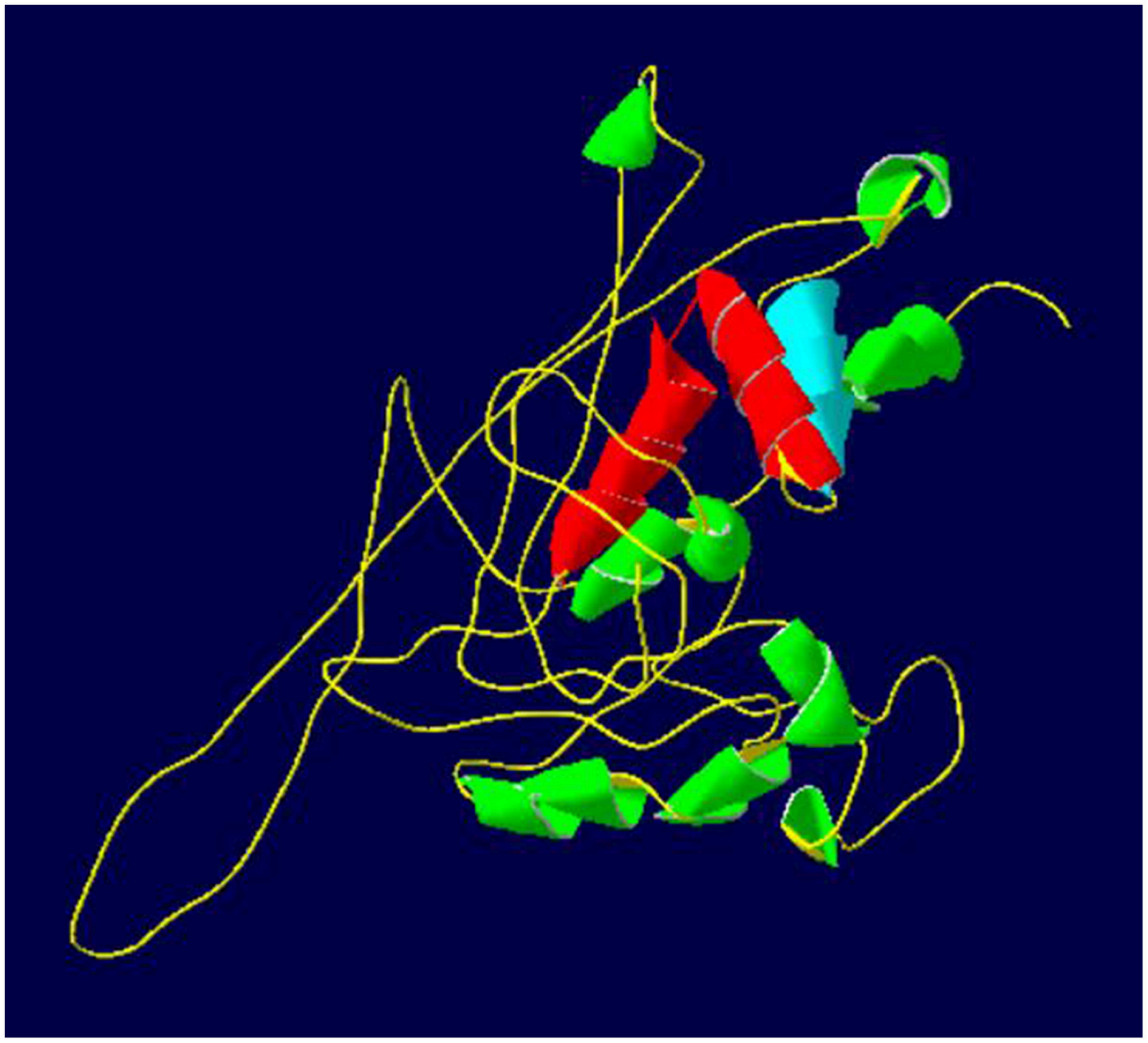
**Predicted tertiary structure of 1R-MYB TF from chickpea**. The repeat (helix-helix-turn-helix structure) and the MYB domain are represented by the blue and red (helix-turn-helix). The highlighted MYB domain region has been predicted based on the 1R-MYB TF from *Solanum tuberosum*. C score for structure: –2.72. The green color represents other helices that are not a part of the MYB domain.

The predicted tertiary structure of 1R-MYB (337 amino acids) TF revealed a DNA-binding domain consisting of one repeat and regularly spaced TRP/hydrophobic residues, [-Trp/hydrophobic residue-(X_19_)-Trp/hydrophobic residue-(X_19_)-Trp/hydrophobic residue]. This single repeat domain consists of three helices (helix-helix-turn-helix), which forms the hydrophobic core for DNA-binding activity (Martin and Paz-Ares, 1997; Ambawat et al., 2013; Figure 2, Supplementary Figure 2).

### Identification of PPI using 1R-MYB TF as the “bait” protein

**Figure 3:**
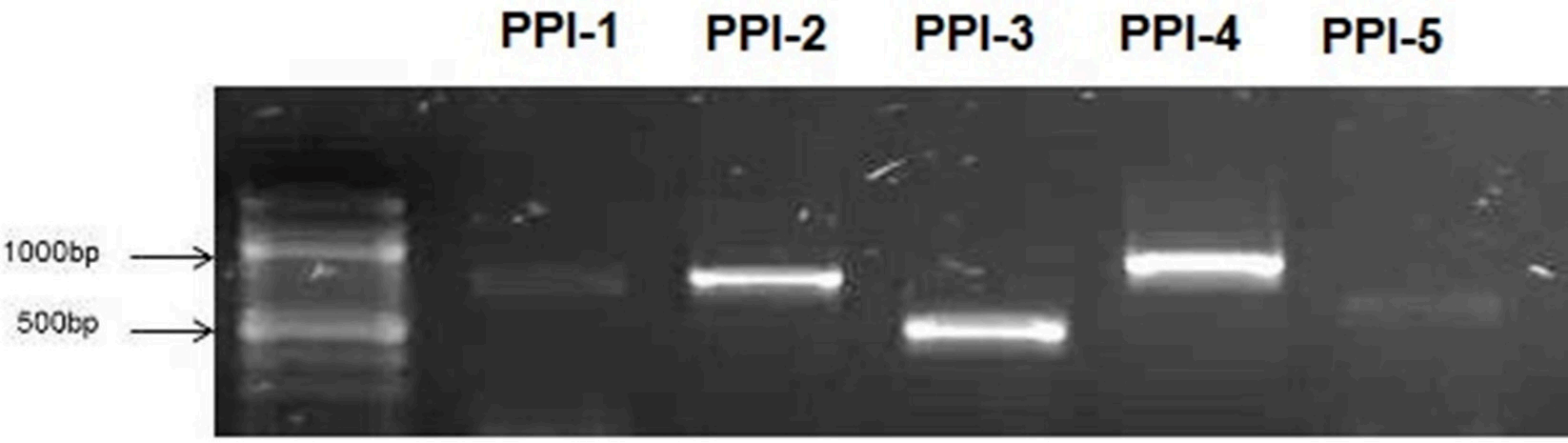
**PCR performed on colonies identified from the QDO/X/A (showing PPI) to identify inserts in prey pGADT7-rec clones)**. PCR performed on the five blue colonies (PPI-1, PPI-2, PPI-3, PPI-4, and PPI-5) identified from the QDO/X/A showed variation in the product length of inserts amplified from pGADT7-Rec.

Diploid cells that expressed proteins with physical interactions were able to grow and form blue colonies on the DDO/X/A by activating resistance to Aureobasidin A (*AbA*^*r*^) and α-galactosidase (α-gal) activity. PCR amplifications from the five blue colonies obtained through screening of the DDO/X/A media, showed distinct amplicon sizes (Figure 3). Patching of the colonies (PPI-1, PPI-2, PPI-3, and PPI-4, PPI-5) on QDO/X/A formed blue growth, indicating the presence of strong PPI causing the activation of all four reporter genes (*AUR1-C, HIS3, ADE2*, and *MEL1*) that were under the control of three completely heterologous Gal4-responsive promoter elements (G1, G2, and M1) (Matchmaker™ Gold Y2H manual; Table 1). Small scale positive and negative PPI control matings showed the expected results when plated on selective media (blue colonies for positive PPI control; and no growth for the negative PPI control on DDO/X/A, see Matchmaker Gold Y2H system User Manual).

**Table 1:** **Determination of PPI strength using biochemical assay**.

**Selective media**	**PPI-1**	**PPI-4**	**PPI-5**
QDO/X/A	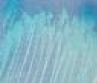	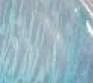	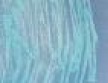
QDO/X/0 mM 3-AT	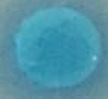	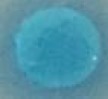	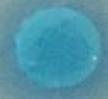
QDO/X/1 mM 3-AT	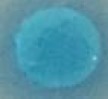	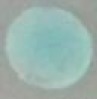	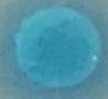
QDO/X/2 mM 3-AT	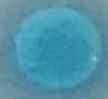	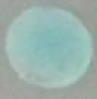	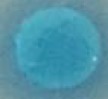
QDO/X/5 mM 3-AT	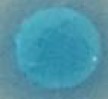	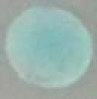	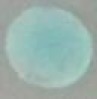
QDO/X/10 mM 3-AT	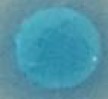	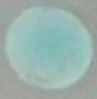	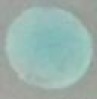
QDO/X/15 mM 3-AT	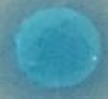	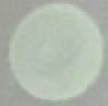	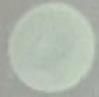
QDO/X/20 mM 3-AT	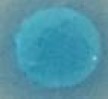	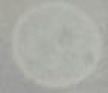	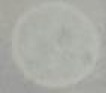

*PPI-1: S. cerevisieae Y2HGold cells transformed with the vectors, bait-1R-MYB and prey- clone 1 (containing partial CDS of GSGT2); PPI-4: S. cerevisieae Y2HGold cells transformed with the vectors, bait-1R-MYB and prey- clone 4 (containing partial CDS of CIPK25); PPI-5: S. cerevisieae Y2HGold cells transformed with the vectors, bait-1R-MYB and prey-clone 5 (containing partial CDS of ABR17-like gene). On the QDO/X/A plates, patching of colonies, PPI-1, PPI-2, PPI-3, PPI-4, and PPI-5 isolated from the DDO/X/A plates showed blue colored growth. Based on sequence analysis of the pGADT7-rec clones, PPI-2 and PPI-3 were omitted from further analysis. Strength of interactions: On plates of QDO/X with varying concentrations of 3-AT (0–20 mM), dark blue spots represent strong interactions. White spots indicate the difficulty in activating the MEL-1 gene that encodes for α-galactosidase in yeast. At 20 mM 3-AT only PPI-1 is able to form blue growth*.

Retransformation of the “bait” clone (*1R-MYB* in pGBKT7) together with the individual “prey” clone of identified interactors in *S. cereviceae* Y2HGold strain confirmed the PPIs detected (blue colonies on the QDO/X/A media). The absence of yeast growth co-transformed with “empty bait” vector and the individual “prey” clones of interactors, on these plates confirmed the absence of false positive interactions.

### Analysis of the 1R-MYB TF interactors

The details of the insert sequences from pGADT7-rec clones for the identified interactors of the 1R-MYB protein, have been summarized in Table 2. The screened sequences were confirmed to be fused to the *GAL4* AD in the correct reading frame. The isolated pGADT7-rec clone 1 from PPI-1 contained a sequence (475 bp) with 99% identity at this region to the CDS of the galactinol-sucrose galactosyltransferase 2 protein (*GSGT2*) from chickpea and encoded a 52 amino acid peptide. In addition, this peptide sequence also showed 77% identity at this region to an alkaline alpha-galactosidase from pea (*Pisum sativum*), and a probable GSGT2 isoform from soybean (*Glycine max*). pGADT7-rec clone 2 from PPI-2 contained a sequence that could not be identified (encoded peptide fused to the GAL 4AD in the incorrect reading frame) while pGADT7-rec clone 3 from PPI-3 contained a contaminant sequence and was omitted from further analysis. pGADT7-rec clone 4 from PPI-4, contained a sequence (572 bp) with 99% identity at this region to the CDS of the CBL (Calcineurin B-like)-interacting serine/threonine-protein kinase 25 (CIPK25) and encoded an 88 amino acid peptide. This peptide sequence also showed 90% identity at this region to a CIPK from medicago. pGADT7-rec clone 5 from PPI-5 was found to contain a sequence (397 bp), with 100% identity to the CDS site of the ABA responsive 17 like protein (ABR17-like) from chickpea, encoding a 46 amino acid peptide. This peptide sequence also shared 91% identity with an ABR17 sequence from medicago and pea, respectively, as well as 79% identity with a pathogenesis related protein, LiPR10.1b from lupin (*Lupinus luteus*). These observations indicate that the proteins isolated were specific to chickpea, although related sequences were also found in other legumes.

**Table 2:** **Details of the pGADT7-rec clones containing CDS encoding peptides fused to the GAL4 AD, identified through Y2H screening with the bait 1R-MYB protein**.

**“Bait” clone in pGBKT7**	**pGADT7-rec clone^a^**	**GenBank accession number**	**Length of peptide (amino acids)**	**Peptide sequence^b^**	**Protein sequence ID**	**Peptide identity^c^**
MYB TF (TUS38128) (complete sequence, 337 amino acids)	1	XM_012713836	52	GKFGVYSSQHPLQCAVDGIDTDFNYDSETGLTTFSIPVPQEGMYRWSIEIQI	XP_012569290	GSGT2
GenBank accession: XM_004508882 Protein sequence accession: XP_004508939.1	4	XM_004498761	88	EIVSKIESAAKSLRFKVGKVKEFKLKLQGMMEGRKGKLAVTAEIYEVAPELAVVEFSKCSGDTFEYVKFFEDDVRPALKDIVWSWQGE	XP_004498818	CIPK25
	5	NM_001309718	46	SIVKISVKYHTKGDLVLSDAVRDETKAKGTGLLKAIEGYVLANPDY	NP_001296647	ABR17-like

a *Prey clone isolated from the pGADT7-Rec library*.

b *Peptide sequence identified (based on BLASTX analysis). The nucleotide sequence encoding the peptides fused to the GAL 4 AD, was confirmed to be in the correct reading frame*.

c *Annotation of sequence is based on both BLASTN and BLASTX analysis*.

### Confirming the PPI strengths for the MYB TF

#### Biochemical assay

The incorporation of 3-AT into the QDO/X media allowed the strength of the PPI to be examined. At 0 mM 3-AT, all three interactors (PPI-1, PPI-4, and PPI-5), showed strong growth on the selective media. At 20 mM 3-AT however, only PPI-1 (*S. cerevisiae* Y2H cells containing the bait-*1R-MYB* and the prey vector with a *GSGT2* gene (partial), showed the ability to form a blue spot (Table 1).

#### qRT-PCR analysis

**Figure 4:**
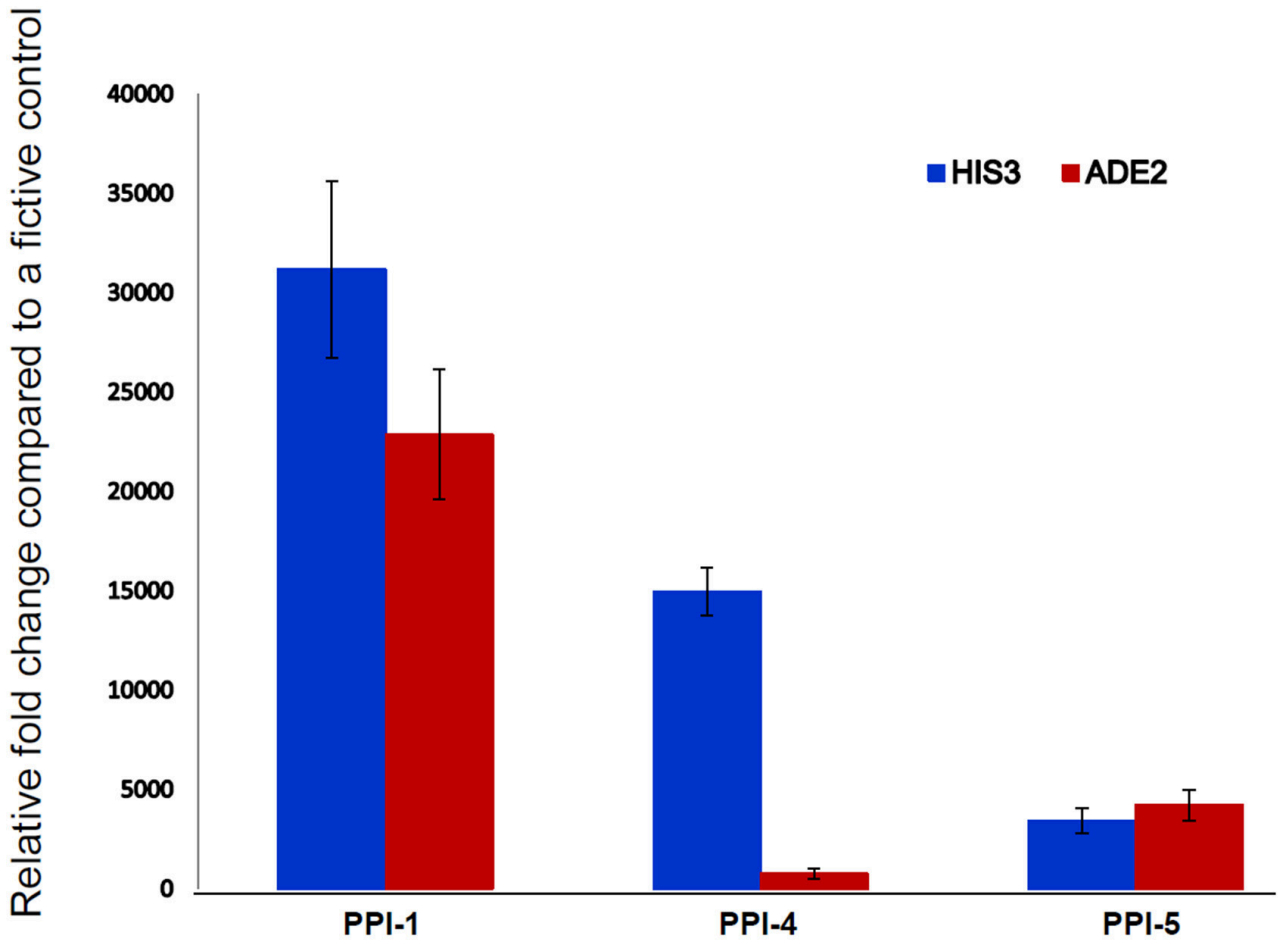
**Confirmation of PPI strength using qRT-PCR**. The qRT-PCR analysis quantified the expression of two reporter genes *HIS3* and *ADE2* for determining the strength of PPI for the selected yeast colonies (PPI-1, PPI-4, and PPI-5). These genes are under the control of Gal4-responsive promoters. The interaction between the bait and prey proteins allow the Gal4-responsive *HIS3* and *ADE2* genes to biosynthesis histidine and adenine for cell growth.

The qRT-PCR assay quantifies the expression of two reporter genes, *HIS3* and *ADE2*, controlled by the Gal-4 responsive promoters. The three interactions from PPI-1, PPI-4, and PPI-5 showed varying relative fold change compared to a fictive control of 35 (Maier et al., 2012), with PPI-1 showing the highest, followed by PPI-4 while PPI-5 showing the least strength of interaction with the 1R-MYB (Figure 4). The qRT-PCR confirms the observations from the biochemical assay in which PPI-1 showed the highest strength of interaction.

## Discussion

### Response to drought stress in chickpea roots

Drought stress has adverse effects on the productivity of chickpea and therefore understanding tolerance mechanisms is essential for knowledge-driven crop improvement. The present study was initiated to understand the mechanism of drought tolerance in chickpea roots. Plant growth and development hugely rely on the root system for uptake of water hence dehydration conditions are sensed early in the root system which further stimulates early signals that affect several processes such as the regulation of root growth, root-shoot signaling, and resource acquisition (Schachtman and Goodger, 2008). Therefore, molecular analysis of drought stress response in roots would provide valuable information regarding key components that are involved in drought responsive signaling mechanisms and tolerance.

Transcriptomics approaches in chickpea have been successfully applied to identify differentially expressed genes in root tissue that were related energy metabolism, TFs, cell signaling, and hormone signaling in response to drought (Molina et al., 2008; Hiremath et al., 2011). Gene expression studies have also identified genes that may play crucial roles in the early signaling for drought tolerance in chickpea root which are valuable information for the identification of candidate genes and biomarkers (Molina et al., 2008; Deokar et al., 2011; De Domenico et al., 2012).

### Putative role of the *MYB* genes in drought stress response

TFs such as APETALA 2, CAP2, and CarNAC3 were suggested to be important mediators of drought stress responses in chickpea (Shukla et al., 2006; Peng et al., 2009). In the present study, a number of TF genes such as *MYB, WRKY*, and *bZIP* were identified to be up-regulated in the roots of the tolerant chickpea genotype. However, the *MYB* group of genes, particularly TUS38128 (later identified as a *1R-MYB*) showed greater expression in the tolerant genotypes, and was found to be enhanced in the line introgressed with the drought tolerance QTL segment, JG11+. Therefore, the *MYB* genes may have a more specific role in regulating drought response in chickpea roots. Previously, a tissue-specific *bZIP TF* was also shown to be significantly involved in response to drought stress in the roots of common bean, (Rodriguez-Uribe and O'Connell, 2006).

*In silico* analysis retrieved 206 *MYB* genes belonging to groups of 1R-MYB/MYB-related, R2R3-MYB, and 3R-MYB in chickpea. The MYB superfamily of TFs is one of the largest, most diverse and important mediators of cell division, developmental and metabolic processes, biotic and abiotic stress responses (Dubos et al., 2010). A large family of *MYB* genes suggested to have various biological functions including legume-specific nodulation were identified from soybean (Du et al., 2012). *MYB* genes have been reported to be expressed in several tissue types including roots, leaves, and stem, under stress conditions such as dehydration, salt, and ABA (Urao et al., 1993; Ingram and Bartels, 1996; Kranz et al., 1998; Shin et al., 2002; Seo et al., 2011). Xiong et al. (2014) reported that *OsMYB48-1*, a novel MYB related TF, was associated with enhanced tolerance to drought, salinity, and the ability to regulate the expression of ABA biosynthesis, early signaling and late responsive genes under drought stress in rice.

### The 1R-MYB TF as a key regulator of drought response

In this study, most of the 206 *MYB* genes identified in chickpea, belonged to the *1R-MYB* group (125 genes), which indicated that they may be functionally significant as regulators. The presence of orthologs of the chickpea *1R-MYB* in model and crop legumes such as lotus, medicago and common bean suggests that these genes are conserved in Fabaceae with possibly similar functional properties. A few studies in lotus have demonstrated the functional relevance of *1R-MYB* genes in roots such as in development and legume nodulation signaling pathway (Volpe et al., 2013; Kang et al., 2014).

The regulatory activity of MYB TFs in recognizing and binding DNA with high affinity and specificity has been suggested to involve PPIs and post-translational modifications (PTM; Dubos et al., 2010). Importantly, the functional domains of the MYB TF consist of a central transactivation domain, and a C-terminal negative regulatory domain, besides the DNA binding domain (DBD) at the N-terminus. DNA-binding TFs, transcriptional co-activators, and proteins that alter the MYB protein's activity, e.g., in PTM have been suggested to interact with MYB proteins (Ness, 1999). In this study, the Y2H approach used to screen a drought stressed chickpea root cDNA library prepared from the tolerant genotype, identified partial CDS encoding for GSGT2, CIPK25, and ABR17-like protein regions as potential interacting partners. Although preliminary, this observation suggests that the expression of the *GSGT2, CIPK25*, and *ABR17-like* genes and the involvement of PPI may have some relevance in chickpea root drought stress regulation.

qRT-PCR analysis of the *CIPK25* gene in the present study (data not shown) showed that it was up-regulated by ~5 folds in the tolerant chickpea genotype. In contrast, subtle expression was observed for GSGT2 and ABR17 in the tolerant genotype compared to the sensitive genotype. A recent study showed that the transgenic expression of this chickpea CIPK25 (XP_004498818), enhanced root growth and tolerance to dehydration and salt stress (Meena et al., 2015).

Serine/threonine kinase and GSGT proteins were shown to be drought-responsive with proteomics approaches (Sharma et al., 2012). The CIPK proteins which are members of the serine-threonine kinases, targeted by CBL, and their genes have been shown to be up-regulated under drought stress in root tissues of chickpea (Li et al., 2009; Wang et al., 2012). Transcriptional analysis of *ABR17* from pea in transgenic Arabidopsis (*Arabidopsis thaliana*), under abiotic stress conditions showed significant changes in the transcript abundance of genes related to stress response, plant growth, and development. Additionally it was also suggested that stress tolerance is mediated by ABR17, a member of the pathogenesis-related (PR) 10 protein, through the modulation of ABA responsive genes (Krishnaswamy et al., 2008). Large scale differential gene expression also showed that the *ABR17* gene was induced in the tolerant chickpea genotype under drought stress (Hiremath et al., 2011). The ability of the same set of genes to be induced by ABA and water stress shows the possibility of “cross-talk” in the regulatory mechanism of stress response (Tuteja, 2007).

The regulation of complex regulatory networks has been proposed to involve multiprotein complex mediated gene expression and transcription regulation (Wolberger, 1999). For example in regulating ABA responsive gene expression, bZIP TFs has been shown to interact with a calcium-dependent protein kinase, CPK32, and DREB2C (Choi et al., 2005; Lee et al., 2010). Previous studies have demonstrated the interacting capability of the MYB proteins and its ability to modulate signal transduction (Zimmermann et al., 2004; Shin et al., 2007). The interaction between the 1R-MYB TF and GSGT2 related protein may have some functional relevance in the regulation of the biosynthesis of sugars in response to stress. The overexpression of the *PAP1* gene encoding a MYB TF in Arabidopsis has been shown to induce the expression of glycosyltransferase genes (Tohge et al., 2005). GSGT has been characterized in faba bean (*Vicia faba*) and was reported to be involved in the synthesis of raffinose with osmoprotectant and antioxidant properties for protecting plant cell walls from oxidative damage (Lehle and Tanner, 1973; Nishizawa-Yokoi et al., 2008). In the osmotic signaling pathways, phosphorylation can be modulated by CIPK and the MYB proteins contain several serine and threonine residues at the C-terminal domains which may act as a substrate for kinases which influence DNA binding activity (Martin and Paz-Ares, 1997; Boudsocq and Laurière, 2005). TFs and kinases have previously been shown to interact in response to stress. For example, the ABA-induced protein kinase PKABA1, a component of the signal transduction pathway that is involved in the ABA-suppressed gene expression was found to interact with an ABA response element-binding factor family of basic leucine zipper TF (ABF) in wheat (Johnson et al., 2002).

**Figure 5:**
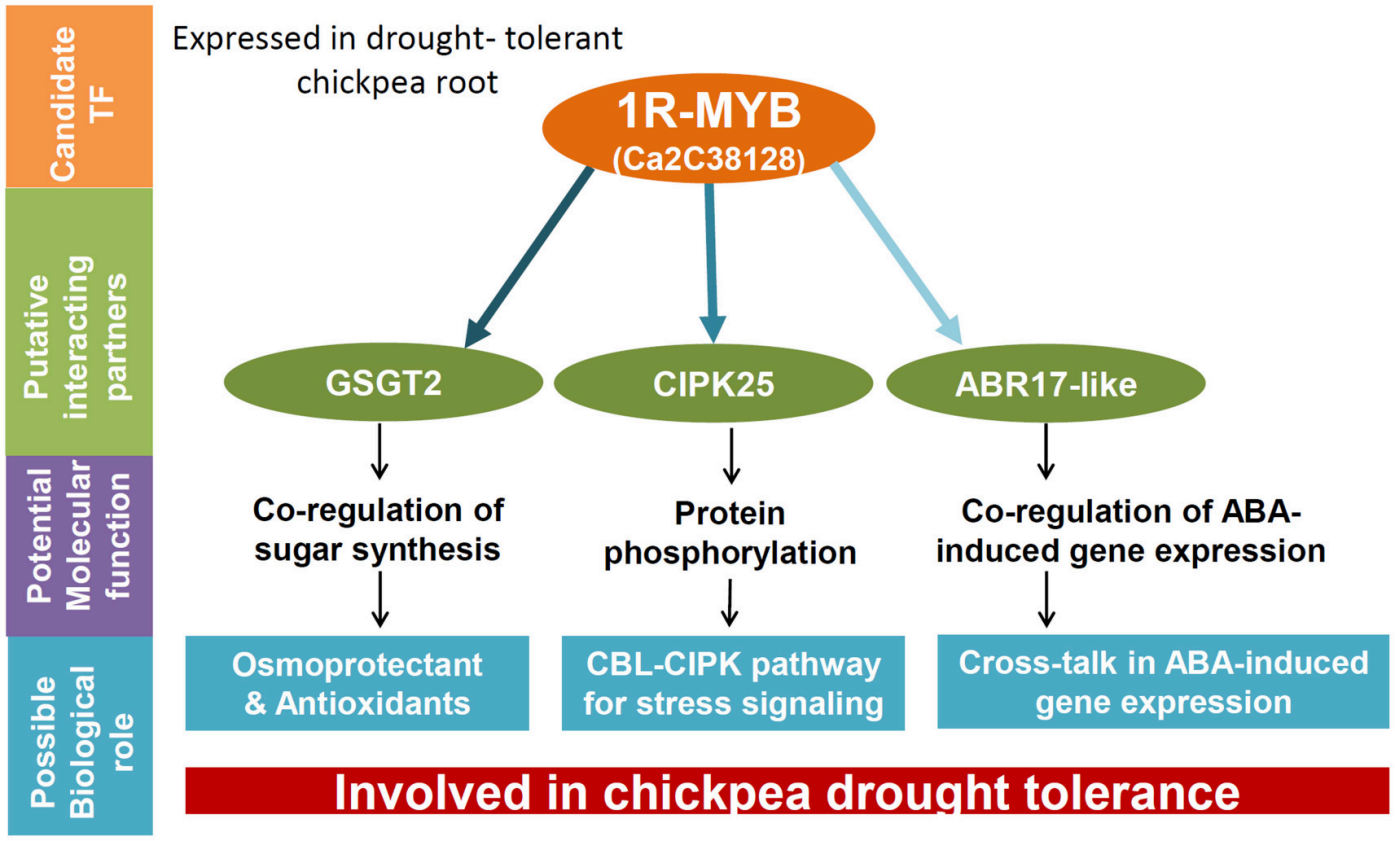
**A hypothetical model proposing a possible role for the 1R-MYB TF in co-regulating drought tolerance in chickpea**. The Y2H analysis involving the candidate TF, 1R-MYB suggests GSGT2, CIPK25, and ABR17-like proteins as possible co-regulators for drought tolerance in chickpea root. These proteins have been known to be involved in various signal transduction and stress responses to drought from previous reports and has been depicted in the figure. The color intensity of the arrows indicate the strength of interactions observed through biochemical and qRT-PCR analyses in the present study.

In summary, the 1R-MYB TF may have an important role in co-regulating drought tolerance in chickpea roots. Since drought conditions are sensed early in the root system, further investigation of this preliminary discovery will be vital for understanding the involvement of MYB TFs in the mechanism of drought response in chickpea. A schematic explanation for the observed PPI involving the 1R-MYB TF in co-regulating drought tolerance is shown in Figure 5.

### Conflict of interest statement

The authors declare that the research was conducted in the absence of any commercial or financial relationships that could be construed as a potential conflict of interest.
