# Supplementary material for: Gene Expression and Yeast Two-Hybrid Studies of 1R-MYB Transcription Factor Mediating Drought Stress Response in Chickpea (Cicer arietinum L.)
Source: Front Plant Sci. 2015 Dec 24;6:1117. doi: 10.3389/fpls.2015.01117 (PMC4689849; doi:10.3389/fpls.2015.01117)
Supplement: Supplementary file 1 [file Table1.DOCX]

**Supplementary Table 1** Primers used for amplification of gene and cDNA library generation

| **Primer name** | **Sequence (5’-3’)** |
| --- | --- |
| ***Gene amplification, cDNA library*** | |
| MYBF | CATGGAGGCC***GAATTC***ATGACCCGACGTTGCTCC |
| MYBR | GCAGGTCGAC***GGATCC***TCAAACT GCATGGATGGCATT |
| CDS 111 | ATTCTAGAGGCCGAGGCGGCCGACATG-d(T)_30_VN |
| SMART 111-modified oligo | AAGCAGTGGTATCAACGCAGAGTGGCCATTATGGCCGGG |
| 5’ PCR Primer | TTCCACCCAAGCAGTGGTATCAACGCAGAGTGG |
| 3’ PCR Primer | GTATCGATGCCCACCCTCTAGAGGCCGAGGCGGCCGACA |
| T7 | TAATACGACTCACTATAGGGC |
| 3’AD | AGATGGTGCACGATGCACAG |
| ***qRT-PCR*** | |
| Ca2S032873_1646_3001F | GAAACTCAAGCTCAGCCGAC |
| Ca2S356591_1831_0898F | CAACTGATCAGACCAATCAAGC |
| Ca2C37998F | GCGAGGAAACAAGTGGAGAG |
| Ca2S107358_1454_0701F | TGTCTCATACGAGCTTCCCA |
| Ca2C41582F | CATCTTCGCACTCTTCTCCC |
| Ca2C161F | GGACAACCGAAGAGGATCAA |
| Ca2S162925_1065_2547F | TGGGAAGAGCTCCTTGTTGT |
| Ca2C38128F | TAAAAACGCGTACCGAATCC |
| Ca2S032873_1646_3001R | TGCAAAGTCATCTGCTTCTGA |
| Ca2S356591_1831_0898R | ACATGTATGGGTGAGGAGCA |
| Ca2C37998R | TTCCCCCATTGGTAAAAACA |
| Ca2S107358_1454_0701R | TGCGGATATCAATTTCAACG |
| Ca2C41582R | GATCAGCAGCAACCACAAGA |
| Ca2C161R | GTTGTCCGTCCTTCCTGGTA |
| Ca2S162925_1065_2547R | TCCCACACCTTAATAATCCTGC |
| Ca2C38128R | GTCGGCTTTTCAAGCAACTC |
| HIS3_F | CAGTAGCAGAACAGGCCACA |
| HIS3_R | TTCAGTGGT GTGATGGTCGT |
| ADE2_F | TATGGCGGAATGTGAACAAA |
| ADE2_R | CAAATGGAACGCCAAAATCT |
| TAF10_F | ATATTCCAGGATCAGGTCTTCCGTAGC |
| TAF10_R | CAACAACAACATCAACAGAATGAGAAGACTAC |
| UBC6_F | GATACTTGGAATCCTGGCTGGTCTGTCTC |
| UBC6_R | GCAAATACAGGTGATGAAACAGAAGACCCTTT |

Underlined nucleotides represent the pGBKT7 vector section. Bold and italicized nucleotides represent *BamH1* and *EcoR1* restriction sites
