# Supplementary figures and images for: Gene Expression and Yeast Two-Hybrid Studies of 1R-MYB Transcription Factor Mediating Drought Stress Response in Chickpea (Cicer arietinum L.)
Source: Front Plant Sci. 2015 Dec 24;6:1117. doi: 10.3389/fpls.2015.01117 (PMC4689849; doi:10.3389/fpls.2015.01117)

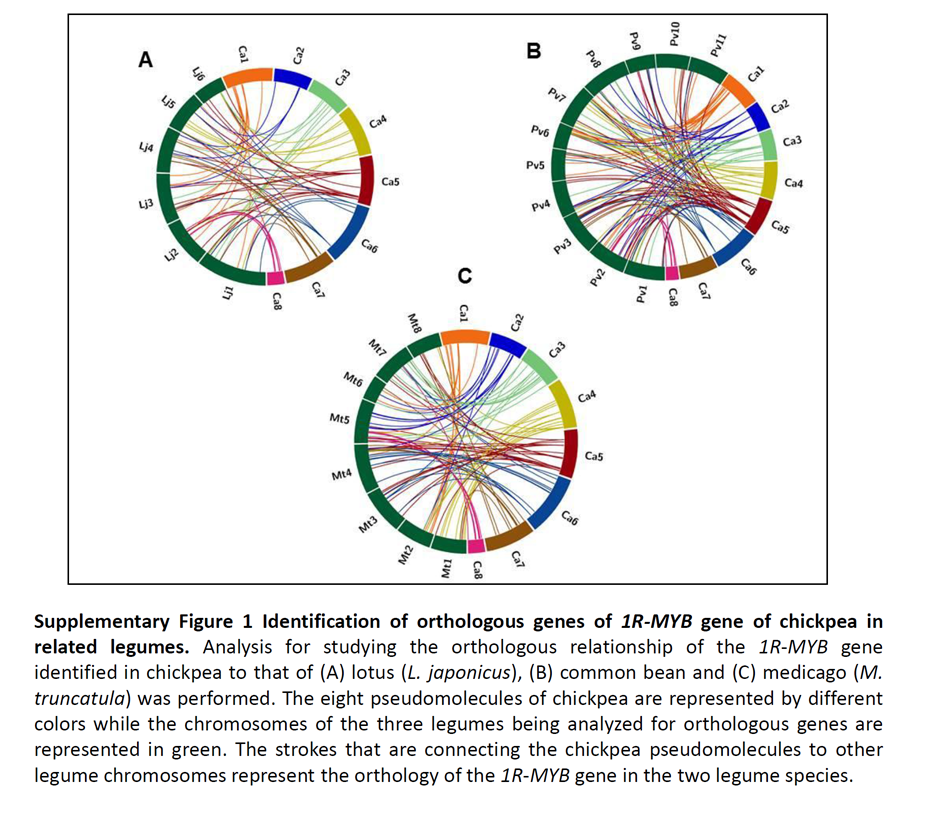

Supplement: Supplementary file 2 [file Image1.tif]

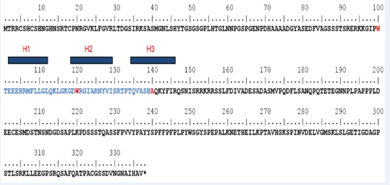

Supplement: Supplementary file 3 [file Image2.tif]
